# Episodic homelessness and health care utilization in a prospective cohort of HIV-infected persons with alcohol problems

**DOI:** 10.1186/1472-6963-6-19

**Published:** 2006-02-27

**Authors:** Theresa W Kim, Stefan G Kertesz, Nicholas J Horton, Nicole Tibbetts, Jeffrey H Samet

**Affiliations:** 1 Clinical Addiction Research and Education (CARE) Unit, Section of General Internal Medicine, Department of Medicine, Boston University School of Medicine, Boston, MA, USA; 2 Division of Preventive Medicine, Department of Medicine, University of Alabama at Birmingham School of Medicine and the Deep South Center on Effectiveness and the Birmingham Veterans' Affairs Medical Center, Birmingham, AL, USA; 3 Department of Mathematics, Smith College, Northampton, MA, USA; 4 DM-STAT Inc., Medford, MA, USA; 5 Department of Social and Behavioral Sciences, Boston University School of Public Health, Boston, MA, USA

## Abstract

**Background:**

Because individuals with HIV/AIDS often have complex medical and social needs, the impact of housing status on medical service utilization is difficult to isolate from the impact of conditions that may worsen during periods of homelessness such as depression and substance abuse. We examine whether episodes of homelessness are independently associated with suboptimal medical utilization even when accounting for concurrent addiction severity and depression.

**Methods:**

We used data from a 30-month cohort of patients with HIV/AIDS and alcohol problems. Housing status, utilization (ambulatory visits, emergency department (ED) visits, and hospitalizations) and other features were assessed with standardized research interviews at 6-month intervals. Multivariable longitudinal regression models calculated incidence rate ratios (IRR) comparing utilization rates during 6-month intervals (homeless versus housed). Additional models assessed whether addiction severity and depressive symptoms could account for utilization differences.

**Results:**

Of the 349 subjects, 139 (39%) reported homelessness at least once during the study period; among these subjects, the median number of nights homeless per 6-month interview period was 30. Homelessness was associated with higher ED utilization (IRR = 2.17; 95% CI = 1.72–2.74) and hospitalizations (IRR = 2.30; 1.70–3.12), despite no difference in ambulatory care utilization (IRR = 1.09; 0.89–1.33). These associations were attenuated but remained significant when adjusting for addiction severity and depressive symptoms.

**Conclusion:**

In patients with HIV/AIDS and alcohol problems, efforts to improve housing stability may help to mitigate intensive medical utilization patterns.

## Background

Indigent populations are disproportionately affected by HIV infection. The prevalence of HIV infection is 5 to 9 times higher in urban poor populations than the general population [1,2]. Approximately one half of individuals with HIV/AIDS who receive services funded by the Ryan White Comprehensive AIDS Resources Emergency Act live below the Federal Poverty Level [3]. In the HIV Cost and Utilization Study (HCSUS), 46% reported an annual income less than $10,000 [4].

In part due to the overlap between poverty and unstable housing, many individuals with HIV infection experience homelessness. About one third of HCSUS participants reported a need for housing services with 39% of those needing housing services unable to access these services [5]. Among HIV-infected veterans, 32% have been homeless at some point in their lives [6] compared to 7.4% of the general population [7]. Prevalence estimates of homelessness in persons with HIV range from 6% to 27% among a New York State Medicaid population [8], clients at federally funded HIV clinics [3], and adults with substance abuse disorders [9]. In comparison, 3.1% of the general population sampled by random digit dialing has been homeless in the past 5 years [7].

Homelessness among HIV-infected persons is associated with a lower likelihood of receiving prophylaxis for opportunistic infections [10] and highly active antiretroviral therapy [11] as well as higher mortality rates [12]. Reduced access to effective therapies occurs in the context of higher rates of emergency department (ED) utilization [9,13] and hospital admissions [8,9]. Because indigent patients with HIV/AIDS often have complex medical and social needs, the impact of housing status is difficult to isolate from other conditions that increase the risk of homelessness and affect access to care such as addictions [9,14-16] and depression [17-19]. Previous studies on the impact of housing status on utilization among individuals with HIV infection have modeled homelessness as a permanent condition despite evidence that most individuals who are homeless have intermittent periods of stable housing [20]. In this study of the relationship between homelessness and health care utilization, we elected to treat homelessness as a state that could vary over time.

To the extent that homelessness might be associated with distinct patterns of health service utilization among HIV-infected individuals, relevant policy decisions ultimately must pivot upon clarification of which particular problems need targeting. That is, high cost utilization patterns could reflect worse HIV progression, greater addiction severity, impaired access to a usual source of ambulatory care, or potentially the impact of homelessness itself. Each of these associations might arguably call for somewhat different policy responses. Hence, a secondary interest of this study is to disentangle, in an exploratory way, contributing factors to utilization patterns.

Among HIV-infected individuals, alcohol misuse demarcates a group at special risk for adverse outcomes [6] including housing instability. We therefore used data from a cohort study of HIV-infected individuals with alcohol problems to examine the hypothesis that HIV-infected persons would utilize less ambulatory care and more emergency department and inpatient care during periods with an episode of homelessness, compared to periods without homelessness. Furthermore we set out to explore the independent contributions of relevant variables (addiction severity and depressive symptoms) with the expectation that these variables would substantially attenuate and potentially explain any apparent association between homelessness and service utilization patterns in this sample of HIV-infected persons with a history of alcohol problems.

## Methods

### Study population

We analyzed data from the HIV-Alcohol Longitudinal Cohort (HIV-ALC) study. The primary purpose of the HIV-ALC study was to prospectively examine the impact of alcohol use on HIV disease progression. Subjects in the HIV-ALC cohort receiving antiretroviral therapy were eligible to participate in a randomized controlled trial of an antiretroviral adherence intervention. A description of the patients in the HIV-ALC cohort [21] as well as the antiretroviral adherence intervention has been previously published [22]. Briefly, eligibility for the HIV-ALC study included endorsement of two or more positive responses to the CAGE questionnaire [23] or a physician co-investigator clinical diagnosis of lifetime alcohol abuse or dependence. The eligibility criteria of a history of alcohol problems was determined by the CAGE questionnaire in 313/349 (90%) of subjects, and based on clinical assessment in 36/349 (10%) of subjects. Other entry criteria included the following: fluency in English or Spanish, Mini-Mental State Examination score greater or equal to 21 [24], and no plans to move from the Boston area in the two years following the baseline assessment. All subjects had HIV infection confirmed either as part of clinical care or as part of the study. Subjects were recruited from the following sites: 56% from the Boston Medical Center HIV Diagnostic Evaluation Unit [25]; 17% from posted flyers; 13% from Boston Medical Center Primary Care Clinic; 5% from a medical respite facility for homeless persons; 4% from a methadone clinic; 4% from subject referrals; and 2% from Beth Israel Deaconess Medical Center.

Of 444 eligible subjects screened, 349 (79%) provided informed consent to participate in the study. Because study subjects were recruited over a four-year period (1997 to 2001), and all follow-ups ceased in August 2001, time of recruitment was the major factor affecting the number of follow-up observations in this study (P < .0001) [26].

Those subjects receiving antiretroviral therapy at the time of recruitment participated in a randomized controlled trial to enhance adherence to antiretroviral therapy. The intervention consisted of three nurse visits over a 3-month period to problem-solve with the patient about ways to decrease missed doses. The intervention was not significantly associated with higher adherence [22].

The Institutional Review Boards of Boston Medical Center and Beth Israel Deaconess Medical Center approved this study.

### Data collection

Subjects were interviewed up to 7 times over the study period, approximately 6 months apart, from 1997 to 2001. At each scheduled interview, trained research associates, using a standardized instrument in either English or Spanish, obtained information about housing status, medical service utilization, HIV risk behaviors, antiretroviral medication use, substance use, addiction severity, and depressive symptoms. The Spanish interview instrument used the standardized Spanish versions of scales when available; the remainder of the questionnaire was translated from English into Spanish, back translated to check for accuracy, and then corrected. CD4 cell counts and HIV RNA levels were collected, using existing laboratory tests if performed as part of clinical care within six months of the interview. When clinical samples were unavailable, the Boston Medical Center Clinical Laboratory evaluated blood samples collected for study purposes.

### Outcome variables

Our three outcomes of interest were the number of self-reported ambulatory visits, the number of emergency department (ED) visits, and the number of hospitalizations in the 6 months prior to the research interview.

### Main predictor variable

We used subjects' report of any night spent on the street or in a shelter in the past 6 months to indicate an episode of homelessness [27]. This was assessed with the survey question, "In the last 6 months, how many nights have you spent in an overnight shelter, or on the street, without shelter?" Sleeping in environments intended for temporary shelter, or in places not meant for sleeping, corresponds to the federal McKinney Act's definition of homelessness and approximates "literal homelessness"[28].

### Other explanatory variables

We used the Behavioral Model for Vulnerable Populations [29] as a conceptual framework to help guide our choice of covariates for inclusion in the multivariable regression models explaining health service use. Predisposing factors (age, gender, race/ethnicity, housing status, substance abuse severity, and depressive symptoms) and relevant indicators of need for medical health service use (CD4 cell count, HIV RNA viral load, receipt of any antiretroviral therapy) were included in the models. Health insurance status, an enabling/disabling factor, was measured but not included in the models since 99% of all subjects had access to private, Medicaid or a special publicly-funded health insurance for medications, ambulatory and ED visits, and hospitalizations. Substance abuse severity was assessed with the alcohol and drug composite scores from the Addiction Severity Index (ASI-alc and ASI-drug, respectively), an assessment instrument with documented reliability and validity, each scored 0–1, with higher scores indicating increased severity [30]. Depressive symptoms were measured with the 20-item Center for Epidemiologic Studies Depression Scale (CES-D); scores ≥ 16 are considered to reflect significant depressive symptoms [31]. Participation in the intervention trial and study time point were also included as potential explanatory variables.

### Analysis

Descriptive statistics (proportions, means, standard deviations) and univariate analyses were used to compare subjects by housing status at baseline. Categorical variables were compared using chi-square test and continuous variables with the two-sample t-test. We calculated the proportion of subjects experiencing homelessness over time using a Kaplan-Meier survival estimator. Since we asked about homelessness in the 6 months prior to each interview, time 0 was considered to be 6 months prior to the first interview, therefore the survival estimator calculated the proportion of subjects experiencing homelessness over 36 months.

To examine the association of homelessness and medical service utilization, we constructed separate multivariate longitudinal regression models for each outcome: ambulatory visits, ED visits, and hospitalizations. The unit of analysis for regression models was by observation (e.g. interview). Longitudinal regression models calculated incidence rate ratios (IRR) for each available 6-month observation period (homeless versus housed). Since serial measures on the same individuals were collected, generalized estimating equation (GEE) regression models[32] were used to adjust for the correlation between these measures over time. We used an empirical working variance estimator in these models and log link function (Poisson regression).

In order to focus on the statistical significance of the homelessness variable (any versus none), we used the Behavioral Model for Vulnerable Populations to build multivariate models with covariates, including predictor variables that proved to not be significant. The following variables were included in all models: age, gender, race/ethnicity (2 df), study time point (6 df), CD4 cell count, HIV RNA log_10 _viral load, receipt of antiretroviral therapy (any or none) and participation in the antiretroviral adherence intervention (adherence intervention group, control group, and not on antiretroviral medication; 2 df). To explore whether the statistical significance of homelessness was affected by inclusion of addiction severity and depressive symptoms in the equations, models were also constructed with variables for alcohol abuse severity (ASI-alc score), drug abuse severity (ASI-drug score), and any depressive symptoms (CES-D ≥ 16). All the predictor variables were allowed to vary with time except for age, gender, race/ethnicity, and intervention trial assignment group.

We performed a secondary analysis to examine whether there was a "dose-response" relationship between the number of nights homeless and utilization differences. We analyzed homelessness based upon the cumulative number of nights homeless in a 6-month observation period. The median number of nights homeless (i.e., 30) and interquartile range (7,90) were used to define the 5-level categorical homelessness variable.

All analyses were run using SAS statistical software version 8.2 [33].

## Results

### Subject characteristics

Descriptive characteristics of the cohort (n = 349) stratified by housing status reported at the first (baseline) interview are presented in Table 1. Compared to housed subjects, more of the subjects reporting homelessness at baseline (101/349, 29%) were male, had not graduated from high school, and had recently been incarcerated, injured, or abused (either physically or sexually). In addition, homeless subjects endorsed significantly more depressive symptoms on the CES-D (27.2 vs. 20.5, P < .0001). While the mean CD4 cell count was not significantly different between homeless and housed subjects (405 vs. 399 cells/μl, respectively, P = .81), a lower proportion of the homeless subjects reported taking any antiretroviral medications in the previous 6 months compared to the housed (47% vs. 64%, P = .001).

No difference was found in the proportion of subjects who drank any alcohol. However, among those who drank any alcohol, the homeless reported higher alcohol consumption and alcohol abuse severity as reflected by drinks per day (5.5 vs. 1.6, P = .03) and ASI-alcohol composite score (P = .009). While no difference was found in cocaine use (21% vs. 25%, P = .36), a higher proportion of the homeless reported any heroin use (16% vs. 8%, P = .04), and higher drug abuse severity as measured by the ASI-drug composite score (P = .05).

Forty-two percent (148/349) of the cohort had a history of homelessness in the 5 years before entering the study (median duration 6 months, interquartile range 3 to 18 months). As mentioned previously, 29% of the cohort reported homelessness at the baseline interview. The median number of nights homeless in the 6 months before the baseline interview was 30 nights with interquartile range of 7 and 120 (possible range 0–180). By the end of 36 months of observation, 39% (136/349) of the study cohort reported homelessness at least once in the preceding 6 months.

Using all observations (n = 1045), medical service utilization during a 6-month period is summarized as follows (median, 75% quartile, range): ambulatory visits (4, 7, 0–180); ED visits (0, 1, 0–15); and hospitalizations (0, 0, range 0–10).

### Medical service utilization and homelessness

#### Ambulatory visits

No significant difference was found in ambulatory visit utilization between homeless and housed periods in the multivariate longitudinal regression model (IRR 1.09; 95% CI 0.89–1.33). Adjusting for alcohol, drug abuse and depressive symptoms did not markedly change these findings (IRR 1.10; CI 0.91–1.32). Other factors associated with higher ambulatory visit utilization included: female gender (IRR 1.44; CI 1.11–1.87), less severe alcohol abuse (IRR 1.92; CI 1.25–2.94 per one point reduction in ASI-alcohol composite score), and lower CD4 cell count (IRR 1.05; CI 1.01–1.10 per 100 reduction in cell count/μl), earlier study time point (df 6, P = 0.003), and participation in the antiretroviral adherence intervention (df 2, P 0.04). Identifying as Hispanic, however, was associated with lower ambulatory visit utilization (IRR 0.63; CI 0.50, 0.77).

#### Emergency department

Homelessness was significantly associated with greater use of the ED (IRR 2.17; CI 1.72–2.74). This finding was slightly attenuated but remained significant when adjusting for alcohol, drug abuse and depressive symptoms (IRR 1.95; CI 1.55–2.45). As presented in Table 3, one to seven homeless nights were not associated greater use of the ED (IRR 1.31; 0.85–2.15). However, there were significant associations between a higher number of nights of homelessness (8–30, 31–120, and 121–180 nights) and ED utilization rates (IRR 1.49, 2.17, and 2.65, respectively). Other significant predictors of higher ED utilization were female gender (IRR 1.50; CI 1.11–2.03) and more depressive symptoms (IRR 1.02; CI 1.01–1.03 per one point increase in CES-D score).

#### Hospitalization

Homelessness was also significantly associated with inpatient hospitalizations (IRR 2.30; CI 1.70–3.12). This finding was attenuated but remained significant after adjusting for alcohol, drug abuse severity and depressive symptoms (IRR 1.90; CI 1.41–2.56). Similar to the analyses of ED utilization rates, one to seven days of homelessness were not associated with hospitalization rate differences. However, hospitalization rate differences were found with 8 to 30 nights homeless (IRR 1.85; 1.17–2.94), 31–120 nights (IRR 1.85; 1.17–2.94) and 121 to 180 nights (IRR 2.88; 1.95–4.25). Other factors significantly associated with higher hospital utilization rates included: lower CD4 cell count (IRR 1.10; CI 1.03–1.16 per 100 reduction in cell count/μl); worse drug abuse severity (IRR 4.38; CI 1.18–16.33 per 1 point increase in ASI-drug composite score), more depressive symptoms (IRR 1.02; CI 1.01–1.03 per one point increase in CES-D score), and earlier study time point (6 df, P = .05).

## Discussion

In this prospective cohort study of individuals with HIV infection and alcohol problems, utilization of ED and hospital inpatient care was significantly higher during periods in which homelessness was experienced. In addition, greater ED and hospital inpatient utilization differences were found with more nights homeless. These utilization findings were not fully attributable to addiction severity or depressive symptoms.

This study's findings are consistent with prior findings that homelessness is associated with higher ED visits [9,13] and hospitalizations [8,34] in individuals with HIV infection. Prior work, however, was unable to disentangle the simultaneous effects of addiction disorders and depressive symptoms, both of which are known to predict physical functioning in homeless HIV-infected individuals [40] and hypothetically could contribute to higher ED and inpatient utilization. Increased use of ED and hospital inpatient services during homeless periods may have occurred for exposure-related conditions [35] or injuries due to victimization [36]. Even though no difference was found in ambulatory utilization, it remains plausible that the daily struggle to meet basic subsistence needs may have been a barrier to accessing outpatient care in a timely manner (e.g. earlier in the course of an acute illness) [4,37] resulting in ED visits or hospitalizations.

It is important to note that this study modeled homelessness as a time varying "state" rather than a "trait". A subject could contribute to utilization incidence rates for the homeless cohort and subsequently to the housed comparison group if that subject did not experience any homelessness in another interview period. This approach suggests that the state of homelessness contributes to higher hospitalizations and ED utilization, rather than unique features of the "homeless" person. Although causality cannot be proven [38], the finding that a higher number of nights homeless was associated with greater ED and hospitalization differences suggests that homelessness contributes to these utilization differences.

A substantial minority of 29% reported homelessness during the 6 months prior to the baseline interview and 39% at any time during the study period. The proportion of homeless subjects in this study likely reflects selective inclusion of HIV-positive persons with a history of alcohol problems. This cohort's higher incidence of homelessness compared to published reports (6 to 11%) [3,8,13] results, in part, from this study's longitudinal study design, which was more likely to capture both the long-term homeless and persons homeless for short periods of time (i.e. the transiently and episodically homeless)[20]. Since a pattern of intermittent access to conventional housing is relatively common among homeless persons [39], cross-sectional studies tend to over-sample chronically homeless persons [7]. Perhaps more importantly, since many of the patients in this study were at-risk for homelessness for a variety of reasons (e.g., low income, recent incarceration, depressive symptoms, and alcohol or drug addiction) residential instability could have resulted from the worsening of just one of these factors for individuals without much of a safety net.

Although previous studies have documented less ambulatory care utilization among homeless persons [9,13], we did not find such a difference between homeless and housed periods. We postulate that this may have been due to a recruitment strategy that drew predominantly from an HIV intake clinic that facilitated primary care linkage [25] as well as access to health insurance in a state that aggressively expanded Medicaid during the study period.

There are several important implications for our findings. First, to the extent that an investment in housing and other services to prevent homelessness among HIV-infected persons would require a major allocation of public resources, the cost of not adopting such a strategy needs to be clarified [40]. Studies of service utilization by severely mentally ill homeless persons suggest that housing costs can be offset by savings realized from hospitals and jails, [41] however similar evaluations of housing HIV-infected persons are lacking.

Second, since this study occurred in a city with relatively generous ambulatory care services for the homeless, an even greater use of ED and inpatient hospital services might be expected in settings where ambulatory care for homeless persons is less accessible [42]. Furthermore, since homeless periods were not associated with lower ambulatory utilization in our sample (perhaps reflecting local supply of these services), it seems unlikely that further expansion of homeless ambulatory care programs would fully address excess ED and hospital utilization. To the extent that statistical adjustments for substance abuse severity minimally altered the effects of homelessness, expanded access to addiction treatment services alone may not be sufficient to mitigate intensive medical utilization patterns.

Our study has several limitations. While it would have been ideal to have a night-by-night account of when homelessness and medical service utilization occurred, our data collection precluded determination of whether homeless nights were concurrent with dates of medical service utilization. Additionally, our objective indicators of medical need (CD4 cell count, HIV viral load, and antiretroviral medication use) may not have fully encompassed physical health, since co-morbid conditions have been increasingly recognized for their impact on HIV-infected individuals [43] and may have contributed to utilization differences. Also, medical service utilization was determined by self-report. However, studies have found self-reported health care use to be a valid measure among HIV-infected individuals [44], homeless persons [45], and drug users [46]. Homeless persons are a heterogeneous population, yet we did not differentiate among subgroups that have varying utilization patterns such as the unsheltered or the chronically homeless [47,48]. Also, we examined a subsample of individuals with HIV/AIDS and unstable housing, namely those with a history of alcohol problems. Our findings may not apply to homeless, HIV-infected individuals without a history of alcohol problems. The data was taken from a randomized trial of an antiretroviral adherence intervention. However, participation in the intervention was not associated with ED or hospitalization differences. Finally, while we did not find differential utilization of ambulatory care services according to housing status, the number of ambulatory visits does not capture important information such as longitudinal provider continuity across visits and the provision of integrated case management services. Both have been associated with lower utilization of ED and hospitalizations in other studies [4,49].

The strengths of this study include its careful assessment of alcohol, drug abuse, and depressive symptoms, information frequently missing in other research on utilization in the homeless HIV-infected. Moreover, its longitudinal nature allowed examination of the episodically homeless persons, a group underrepresented in cross-sectional homeless studies [50].

In summary, in HIV-infected persons with alcohol problems, homelessness was associated with higher utilization of ED and inpatient hospitalizations, despite no difference in ambulatory visit utilization. These utilization differences were not fully attributable to alcohol, drug abuse or depressive symptoms. Even a transient episode of homelessness has potentially costly implications for health care utilization among HIV-infected persons with a history of alcohol abuse.

## List of abbreviations

ED: emergency department

IRR: incidence rate ratio

CI: confidence interval

HIV Cost and Utilization Study: HCSUS

HIV-Alcohol Longitudinal Cohort: HIV-ALC

ASI: Addiction Severity Index

ASI-alc: Addiction Severity Index, alcohol composite score

ASI-drug: Addiction Severity Index, drug composite score

CES-D: Centers for Epidemiologic Studies Depression Scale

## Competing interests

The author(s) declare that they have no competing interests.

## Authors' contributions

Theresa W. Kim (TWK)

Stefan G. Kertesz, (SGK)

Nicholas J. Horton (NJH)

Nicole Tibbetts (NT)

Jeffrey H. Samet (JHS)

Conceptualization of study: TK, SGK, NJH, JHS

NJH designed the statistical analysis.

NT performed the statistical analysis.

TWK drafted the original manuscript.

All authors participated in data interpretation, critical revisions of the manuscript, and gave final approval of the submitted manuscript.

## Pre-publication history

The pre-publication history for this paper can be accessed here:



## Figures and Tables

**Table 1:** Demographic and Clinical Characteristics of Study Cohort (N = 349)Stratified by Housing Status at Baseline *

Categorical measures, %	Homeless N = 101	Not homeless N = 248	p-value
Female	13	24	0.02
Race/ethnicity^†^			0.29
Black	38	47	
Hispanic	26	21	
White	37	32	
High school graduate	49	65	0.005
Health insurance^‡^	98	99.6	0.15
Prescribed antiretroviral medications^§^	47	64	0.001
Physical injury^§^	17	9	0.04
Physical or sexual abuse			
Lifetime	78	82	0.40
Recent ^§^	14	7	0.05
Jail ^§^	36	26	0.05
Substance use^||^			
Alcohol	45	42	0.60
Cocaine	21	25	0.36
Heroin	16	8	0.04
Continuous measures, N [std]			
Age	40.8 [6.8]	40.5 [7.5]	0.70
CD4 cell count ^¶^	405 [291]	399 [273]	0.90
HIV RNA (log_10_) ^¶^	47K [95K]	26K [68K]	0.05
Depressive symptoms **	27.2 [14.13]	20.2 [12.08]	< 0.0001
Addiction Severity Index: ^††^			
Alcohol composite score	0.25 [0.25]	0.18 [0.20]	0.009
Drug composite score	0.13 [0.12]	0.11 [0.10]	0.05

* The majority (56%)of study participants were recruited through from the Boston Medical Center HIV Diagnostic Evaluation Unit. The remaining from posted flyers 17%; Boston Medical Center Primary Care Clinic 13%; respite facility for homeless persons 5%; methadone clinic 4%; subject referrals 4%; and Beth Israel Deaconess Medical Center 2%.

^† ^Categories are not mutually exclusive, so total number will >100%

^‡ ^Private, Medicare, Medicaid, or special publicly funded health insurance for individuals with HIV-infection

^§ ^Previous 6 months

^||^Previous 30 days

^¶ ^Laboratory tests collected as part of clinical care were used if performed within six months of the interview. When clinical samples were unavailable, the Boston Medical Center Clinical Laboratory evaluated blood samples collected for study purposes.

** Depressive symptoms were measured with the 20-item the Center for Epidemiologic Studies Depression Scale

^†† ^Range of possible scores 0–1 with a higher score indicating worse addiction severity.

**Table 2:** Multivariable Longitudinal Regression Results of Predictors of Medical Service Utilization Among Adults with HIV Infection and Alcohol Problems (n= 1045 observations)

		**I**RR^† ^(95% CI)	
	Ambulatory	Emergency Room	Hospitalization
Age	1.01 (1.00, 1.03)	1.00 (0.98, 1.01)	1.02 (1.00, 1.04)
Female	1.44 (1.11, 1.87)*	1.50 (1.11, 2.03)**	1.64 (1.11, 2.42)*
Race/ethnicity			
Black	0.86 (0.68, 1.08)	0.99 (0.74, 1.31)	1.30 (0.87, 1.92)
Hispanic	0.63 (0.50, 0.77)***	0.81 (0.56, 1.18)	0.73 (0.44, 1.23)
White	1	1	1
Lower CD4 cell count ^‡^	1.05 (1.01–1.10)*	1.07 (0.98, 1.20)	1.10 (1.03, 1.16)**
HIV RNA viral load log_10_	1.00 (0.94, 1.05)	1.00 (0.94, 1.07)	1.01 (0.92, 1.10)
Antiretroviral medication use	1.06 (0.86, 1.31)	0.91 (0.72, 1.15)	1.00 (0.72, 1.39)
Adherence intervention randomization assignment			
Intervention group	1.28 (1.01, 1.62)*	1.11 (0.82, 1.51)	1.46 (0.99, 2.16)
Control group	1.32 (1.04, 1.68)*	0.88 (0.65, 1.19)	1.07 (0.73, 1.57)
Not a participant ^§^	1	1	1
Alcohol addiction severity (ASI-alcohol)^||^	1.92 (1.25, 2.94)**	1.13 (0.67, 1.93)	1.54 (0.69, 3.44)
Drug addiction severity (ASI-drug) ^||^	1.47 (0.59, 3.64)	2.29 (0.87, 6.06)	4.38 (1.18, 16.33)*
Depressive symptoms (CES-D)	1.01 (1.00, 1.01)	1.02 (1.01, 1.03) **	1.02 (1.01, 1.03)***

Homelessness (any versus none)	1.10 (0.91, 1.32)	1.95 (1.55, 2.45)***	1.90 (1.41, 2.57)***

* P < 0.05

** P < 0.05

*** P < 0.005

^† ^IRR: incidence rate ratio of medical service utilization for 6-month observation period in which homelessness is reported compared to intervals without homelessness. Separate multivariate regression models were constructed for each utilization outcome. Later study time point was associated with lower ambulatory visit utilization rates (df 6, P = 0.003) and lower hospitalization utilization rates (6 df, P = .05). Study time point was not significantly associated with emergency room utilization rate differences.

^‡ ^Per 100 reduction in CD4 cell count

^§ ^Subjects not receiving antiretroviral medications did not participate in the adherence intervention study.

^|| ^Per one point higher score

**Table 3:** Multivariable Longitudinal Regression Results of the Cumulative Number Nights Homeless and Medical Service Utilization (n= 1045 observations).

	IRR^† ^(95% CI)		
Number nights homeless	Ambulatory	Emergency Room	Hospitalization
0	1	1	1
1–7	1.24 (0.90, 1.71)	1.31 (0.80, 2.15)	1.07 (0.64, 1.80)
8–30	1.15 (0.84, 1.57)	1.49 (1.02, 2.19)*	1.83 (1.01, 3.32)*
31–120	1.27 (0.97, 1.67)	2.17 (1.54, 3.07) *	1.85 (1.17, 2.94)**
121–180	0.79 (0.61, 1.03)	2.65 (1.94, 3.61) ***	2.88 (1.95, 4.25) ***

^† ^IRR: incidence rate ratio of medical service utilization for 6-month observations periods Separate multivariate regression models were used for each utilization outcome. All models include age, gender, race/ethnicity (2 df), CD4 cell, HIV RNA viral load log_10_, antiretroviral medication use, adherence intervention participation, presence of depressive symptoms (Center for Epidemiologic Studies Depression Scale), and addiction severity (Addiction Severity Index, alcohol and drug composite scores).

^‡ ^Number nights homeless was defined by the survey question, "In the last six months, how many nights have you spent in an overnight shelter, on the street, without shelter?" Homelessness was categorized based upon the median nights homeless for the sample (i.e., 30) and interquartile range (i.e., 7, 120).
